# Psychosocial work conditions and quality of life among primary health care employees: a cross sectional study

**DOI:** 10.1186/1477-7525-12-72

**Published:** 2014-05-15

**Authors:** Mariza Alves Barbosa Teles, Mirna Rossi Barbosa, Andréa Maria Duarte Vargas, Viviane Elizângela Gomes, Efigênia Ferreira e Ferreira, Andréa Maria Eleutério de Barros Lima Martins, Raquel Conceição Ferreira

**Affiliations:** 1 Estadual University of Montes Claros. Campus Universitário Professor Darcy Ribeiro, Vila Mauricéia, Montes Claros, MG, Brazil; 2 Federal University of Minas Gerais, 6627 Antônio Carlos Avenue, Pampulha, Belo Horizonte, MG, Brazil

**Keywords:** Quality of life, Primary Health Care, Unified Health System, Workers, Professional burnout, Occupational health

## Abstract

**Background:**

Workers in Primary Health Care are often exposed to stressful conditions at work. This study investigated the association between adverse psychosocial work conditions and poor quality of life among Primary Health Care workers.

**Methods:**

This cross-sectional study included all 797 Primary Health Care workers of a medium-sized city, Brazil: doctors, nurses, nursing technicians and nursing assistants, dentists, oral health technicians, and auxiliary oral hygienists, and community health workers. Data were collected by interviews. Quality of life was assessed using the WHOQOL-BREF; general quality of life, as well as the physical, psychological, social and environmental domains were considered, with scores from 0 to 100. Higher scores indicate a better quality of life. Poor quality of life was defined by the lowest quartiles of the WHOQOL score distributions for each of the domains. Adverse psychosocial work conditions were investigated by the *Effort-Reward Imbalance* model. Associations were verified using multiple logistic regression.

**Results:**

Poor quality of life was observed in 117 (15.4%) workers. Workers with imbalanced effort-reward (high effort/low reward) had an increased probability of general poor quality of life (OR = 1.91; 1.07–3.42), and in the physical (OR = 1.62; 1.02–2.66), and environmental (OR = 2.39; 1.37–4.16) domains; those with low effort/low reward demonstrated a greater probability of poor quality of life in the social domain (OR = 1.82; 1.00–3.30). Workers with overcommitment at work had an increased likelihood of poor quality of life in the physical (OR = 1.55, 1.06–2.26) and environmental (OR = 1.69; 1.08–2.65) domains. These associations were independent of individual characteristics, job characteristics, lifestyle, perception of general health, or psychological and biological functions.

**Conclusions:**

There is an association between adverse psychosocial work conditions and poor quality of life among Primary Health Care workers.

## Abstract (pt)

**Resumo:**

**Introdução**

Os trabalhadores da Atenção Primária à Saúde estão frequentemente expostos a estresse no trabalho. Esse estudo avaliou a associação entre condições estressantes no trabalho e qualidade de vida ruim entre trabalhadores da Atenção Primária à Saúde, Minas Gerais, Brasil.

**Métodos**

Trata-se de um estudo transversal analítico, que incluiu os 797 trabalhadores da Atenção Primária à Saúde. A qualidade de vida foi medida pelo WHOQOL-*bref*; considerou-se a questão de ordem geral e os domínios físico, psicológico, social e ambiente, com escores de 0 a 100, quanto maior o escore melhor a qualidade de vida. Os trabalhadores foram considerados com qualidade de vida ruim quando apresentaram escores no menor quartil de cada um dos domínios. O modelo *Effort-reward Imbalance* foi empregado para avaliação de condições estressantes no trabalho; composto pelo componente extrínseco (esforço e recompensa) e intrínseco (comprometimento excessivo no trabalho)*.* As associações foram verificadas por meio de regressão logística múltipla.

**Resultados**

Qualidade de vida ruim foi observada em 15.4% dos trabalhadores. Trabalhadores com desequilíbrio esforço/recompensa (alto esforço/baixa recompensa) apresentaram maior chance de qualidade de vida ruim geral (OR = 1.91; 1.07-3.42), nos domínios físico (OR = 1.62; 1.02-2.66) e ambiente (OR = 2.39; 1.37-4.16); aqueles com baixo esforço/baixa recompensa apresentaram maior chance de qualidade de vida ruim no domínio social (OR = 1.82; 1.00-3.30). Trabalhadores com alto comprometimento no trabalho apresentaram maior chance de qualidade de vida ruim nos domínios físico (OR = 1.55; 1.06-2.26) e ambiente (OR = 1.69; 1.08-2.65). Essas associações foram independentes de características individuais, relacionadas ao trabalho, do estilo de vida, da percepção de saúde geral, das funções psicológica e biológica.

**Conclusões**

Há uma associação entre estresse no trabalho e a qualidade de vida entre os trabalhadores da Atenção Primária à Saúde.

## Background

Quality of life is a multidimensional construct, which can be influenced by aspects of work and personal life, physical and psychological health, social relations, and the environment where a person lives [1]. The World Health Organization conceptualized quality of life as the ‘individual’s perception of their position in life in the context of culture and value systems in which they live, and in relation to their goals, expectations, standards, and concerns’ [2].

Quality of life has been recognized as a relevant measure in various healthy populations, including workers [1]. Work is a social activity and can affect health and quality of life positively or negatively [3].

Job stress has become a subject of interest due to its significant impact on health, and has been associated with various health disorders, such as psychological difficulties, coronary heart disease, signs and symptoms of muscle and skeletal problems, and weight loss or gain [4-8]. A systematic review concludes that flexible working conditions that increase worker control and choice are likely to have a positive effect on health outcomes [9]. Stress has been associated with anxiety and depression, and anxiety and depression are associated with poor quality of life [10-12].

The *Effort-Reward Imbalance* model was developed to analyse job stress. It focuses on reciprocity of exchange in occupational life, in which a perceived imbalance between high effort spent at work and low reward may elicit sustained stress reactions with emotional disorders and adverse consequences for health [13]. Two different sources of effort at work have been defined in this model, namely, extrinsic and intrinsic. The extrinsic refers to time pressure, frequent interruptions, numerous responsibilities, increased workload, and mandatory overtime. Extrinsic rewards refer to respect and esteem, money, career opportunities, and job security [13]. With regard to intrinsic or personal components, a specific pattern of coping with job demands and of eliciting rewards, termed ‘overcommitment’, is introduced. This pattern of coping defines a set of attitudes and behaviours that reflect striving, in combination with a strong desire for approval and esteem [7,13]. People characterized by overcommitment tend to exaggerate their effort. According to the theoretical *Effort-Reward Imbalance* model, overcommitment is assumed to modify (i.e. increase) the deleterious effect of the effort-reward imbalance on health [13].

Studies on stress and quality of life have consistently reported the association between these two conditions. Exposure to adverse psychosocial work environments, measured by the Demand-Control and the *Effort-Reward Imbalance* models, was associated with poor quality of life in various occupations: financial services workers [14], male automotive assembly workers in Malaysia [10], workers in airplane manufacturing plants in southern Germany [15], employees from a manufacturing plant in Japan [16], nursing providers in a university hospital [17], and healthcare workers in military hospitals in Taiwan [18]. Studies about quality of life among healthcare workers were identified in Brazil [17,19-21], three of which were conducted with workers of the public health service [19-21]. Only one of these studies included job stress among its independent variables, which was evaluated using the Demand-Control model [21]. In this study, high pressure at work was associated with poor quality of life in psychological, social and environmental domains [21].

The Family Health Strategy is the chosen strategy of the Primary Health Care organization in the Brazilian public health system, Unified Health System [22]. The Unified Health System consists of a regionalized and hierarchical network, with three levels of care: primary, secondary, and tertiary care [23]. Multidisciplinary teams, which include doctors, nurses, community health workers, auxiliary nurses, dentists, technicians, and auxiliary oral hygienists, work in the Family Health Strategy. The Primary Health Care workers have the following duties in addition to their specific functions: the development of integral and continuous actions for a population, with a focus on health promotion and prevention of injuries, and on healing and rehabilitation [23]. The Family Health Strategy proposes a new way of doing things, based on ‘challenging’ teamwork [24], and overcomes the clinical/biological/Flexnerian model. It has become a strategy used by municipal managers for the reorganization of Primary Health Care.

To achieve quality in primary health care, a major challenge is the labour process among workers [25]. For the provision of satisfactory public health care, it is necessary to have satisfied employees who enjoy a high quality of life. This study hypothesizes that Primary Health Care workers who are exposed to adverse psychosocial working conditions, an effort-reward imbalance, and overcommitment, report a poorer health-related quality of life. This study aimed to examine the association between adverse psychosocial work conditions and quality of life among Primary Health Care workers of the large municipality of Minas Gerais, Brazil.

## Methods

An analytical cross-sectional study, conducted between August and December 2010, included all 797 Primary Health Care workers: physicians, nurses, dentists, nursing technicians, oral health assistants and oral health technicians, and community health workers. Data were collected through interviews. Quality of life was measured using the WHOQOL-BREF, a version validated in Brazil [2], with 24 questions related to four domains of quality of life (physical, psychological, environmental, and social), and two general questions. Each domain contains questions with response options on a *Likert-type* scale, measuring intensity (none to extremely), capacity (nothing to fully), frequency (never to always), and evaluation (very satisfied to very dissatisfied and very bad to very good).

According to the World Health Organization guidelines [26], the scores from each domain of the WHOQOL-BREF were transformed into scores from 0 to 100, with higher scores indicating a better quality of life. Due to the asymmetric distribution, the final scores for each domain were dichotomized according to the lowest quartile. The workers who had lower scores (in the lowest quartile) were considered to have poor quality of life. A similar approach was used with the instrument *Medical Outcomes Study Short-Form General Health Survey* (SF-12) [14]. Questionnaires with 20% or more of unanswered WHOQOL-BREF questions were excluded [26]. Cronbach’s alpha for the WHOQOL-BREF was 0.82.

The general quality of life was obtained from the question of the WHOQOL-BREF, ‘How would you rate your quality of life?’ There were five response options: very poor, poor, neither poor nor good, good, and very good. The responses were grouped into two levels: good quality of life (very good and good) and poor (neither poor nor good, poor, and very poor).

Psychosocial working conditions were assessed by the *Effort-Reward Imbalance* scale, consisting of 23 questions [27], covering the two extrinsic components (6 effort and 11 reward items) and the intrinsic component (6 overcommitment items). For questions regarding effort and reward, the participants who agreed with the items chose one of four options: not at all stressed, a little stressed, stressed, or very stressed. The response options for the overcommitment component were strongly disagree, disagree, agree, and strongly agree. The total scores for each of the components (effort, reward, and overcommitment) were obtained separately from the sum of the scores obtained for each question and then categorized by tertiles [14]. For the effort and overcommitment components, the highest values of the scores represented high effort and high overcommitment, respectively. For the reward component, the higher scores represented low reward. For all three components, the highest score represented more adverse psychosocial working conditions. The categorical variables effort and reward were combined to create an additional variable, effort-reward imbalance, with four ordinal categories: 1) low effort, low reward; 2) high effort, high reward; 3) low effort, high reward; and 4) high effort, low reward. The *Cronbach's Alpha* coefficients for the *Effort-Reward Imbalance* scale were 0.76, 0.73, and 0.78 for effort, reward and overcommitment, respectively.

The other independent variables investigated were

– Individual characteristics: sex, age, years of study, monthly income, marital status (with partner or without partner).

– Job characteristics: work position [superior (physicians, dentists, and nurses), technical (oral health assistant, oral health technician, and nursing technician) or middle (community health agent)], length of time in the profession, length of time working in the Primary Health Care, labour regime [contracted (worker has no job security) or effective (worker has job security)], having another job (yes or no), and job satisfaction. Job satisfaction was assessed by the question, ‘*How satisfied are you with your job?*’ with response options on a *Likert-type* scale: very satisfied, satisfied, moderately satisfied, dissatisfied and highly dissatisfied. The responses were grouped further into three levels: satisfied (very satisfied and satisfied), moderately satisfied, or dissatisfied (dissatisfied and highly dissatisfied).

– Lifestyle: Level of physical activity, smoking habits, and alcohol consumption. Level of physical activity was measured by the International Physical Activity Questionnaire (IPAQ), tested and validated in Brazil [28], which was analysed according to the guidelines of the Center for Laboratory Studies of Physical Fitness in São Caetano do Sul, classifying workers as very active, active, irregularly active, and sedentary. In the data analysis, the last two categories were grouped together, representing the lowest level of physical activity.

– As for smoking habits, for those who reported being smokers or former smokers, the cumulative lifetime use of cigarettes was calculated, considering the daily intensity and duration of smoking throughout the worker’s entire life [29]. The number of packs (20 cigarettes) smoked per day was multiplied by the years of smoking. The categories were defined as light (up to 20 packs/year), moderate (20.1 to 40 packs/year), and heavy (more than 40 packs/year).

 In the case of alcohol consumption, the consumption of beer, wine, and spirits each was considered, based on the answer to the question, ‘What alcoholic beverage do you drink the most?’ To estimate the intensity of lifetime alcohol consumption, a weighted score was calculated taking into account the measurement of the drink (weights of 0.5 for shot, 1 for cup and glass, and 4 for bottle), the quantity of this measurement that was consumed (one, two, three, etc.), the frequency (weights of 0.5 for a frequency of less than once per year, 1 for once per year, 12 for once per month, 52 for once per week, and 365 for once per day), and the number of years of alcohol consumption. To classify the subjects’ alcohol consumption, three categories were considered: never drank; lesser or greater alcohol consumption throughout life (based on median of the score). In the case of wine, consumption was at most 1.71 glasses per day. Due to the low consumption, this variable was dichotomized between those who consume wine or not.

– Health condition: *Perception of general health*: self-perceived health (excellent, good, fair, poor or very poor), dichotomized as positive (very good, good) or negative (fair, poor or very poor).

### Psychological function

Presence of common mental disorders, assessed by the General Health Questionnaire (QSG-12) [30], whose response options were a range of frequencies which people feel in relation to the evaluated aspects. For analysis, the 0011 scoring system was used, with the value 1 indicating higher frequency of negative feelings. All scores were summed, ranging from 0 to 12. The cut-off of ¾ was used to define individuals with or without common mental disorders, respectively [31]. Cronbach’s alpha coefficient for the QSG was 0.79.

### Biological function

Presence of systemic diseases diagnosed by a physician.

The program SPSS® version 17.0 for Windows was used for data analysis. Descriptive statistics were performed to obtain absolute and relative frequencies of the categorical and ordinal variables, and median, mean, and standard deviation for the quantitative variables. The frequency of workers’ responses to each question from the WHOQOL-BREF was obtained for the different levels of the variables effort-reward imbalance, and overcommitment at work. In the bivariate analysis, the association between the independent variables and the general quality of life and each of its domains was verified by simple logistic regression analysis, obtaining a crude odds ratio, its statistical significance, and 95% confidence interval. Variables associated with general quality of life or the domains of quality of life (p < 0.20) in the bivariate analysis, or theoretically important variables in determining this outcome were included in the multiple logistic regression model. The interaction between the categorical independent variables was observed by combining two variables to create a third. Of the new variables, one remained statistically significant in its association with quality of life and its domains, the combination of self-perceived health and mental disorders with four categories (positive self-perceived health and absence of common mental disorders; positive self-perceived health and presence of common mental disorders; negative self-perceived health and absence of common mental disorders; or negative self-perceived health and presence of common mental disorders). This combined variable was used in all models, rather than the isolated use of the two variables that comprise it. Interaction terms between the variables were created between the quantitative variables. The fit of the model was checked using the *Hosmer-Lemeshow* test.

This study was approved by the Research Ethics Committee of Funorte and all participants signed an informed consent form (CEP/SOEBRAS: 0208/08).

## Results

A total of 762 workers participated in the interviews (95.6%); two were excluded because they did not respond to two or more items from the psychological domain of quality of life. The mean age of workers was 31.87 years old (±8.18 years). They studied 13.29 years on average (±3.23 years), had mean monthly income of US$ 715.06 (±US$ 891.53). Most workers were female (79.9%), lived with a partner (51.1%), were contracted (56.2%), and had no other job (84.1%). The middle level contained the most workers (63.0%), followed by superior (21.3%), and technical (15.7%). Approximately half were moderately satisfied with their job (53.4%), 20.5% were dissatisfied, and 26.1% were satisfied. The distribution of workers regarding lifestyle variables were for physical activity level: very active, 29.5%; active, 48.7%; and irregularly active or inactive, 21.8%; smoking habits: non-smokers, 93.3%; light or moderate smokers, 2.0%; heavy smokers, 4.7%; and beer consumption: none, 68.0%; low or moderate consumption, 16.3%; high or very high, 15.7%. Most did not drink wine (92.4%) or spirits (97.4%); 91.6% of workers showed positive self-perceived health; 29.9% had an illness diagnosed by a physician and 22.4% exhibited common mental disorders.

Most workers reported having good quality of life (84.6%); 117 workers (15.4%) related poor quality of life. The prevalence of poor quality of life in the physical domain was 37.1%; 25.1% in the psychological domain; 21.9% in the social domain, and 27.2% in the environment domain. The 25th percentiles scores for quality of life domains were the following: physical 25th percentile = 64; psychological 25th percentile = 60; social 25th percentile = 67; environmental 25th percentile = 50.

More than half of the workers did not report adverse psychosocial work conditions, as represented by low effort/high reward (49.2%); 13.9% demonstrated high effort/high reward; 12.2% low effort/low reward; 24.6% high effort/low reward, and 40.5% reported overcommitment at work. The frequency of poor quality of life for all items that comprise the domains of quality of life according to the different levels of the effort-reward imbalance variable and overcommitment at work is presented in Table 1.

**Table 1:** **Comparison between the frequencies**^ϕ^** of poor quality of life according to effort/reward unbalance and overcommitment**

	**Low effort, high reward**	**High effort, high reward**	**Low effort, low reward**	**High effort, low reward**	**p value**^**¥**^	**Overcommitment**		**P value**^**¥**^
						**High**	**Low**	
	**(n = 348)**	**(n = 98)**	**(n = 85)**	**(n = 174)**				
***Physical domain***								
Physical pain prevents you from doing what you need to do	332 (95.4)	94 (95.9)	84 (98.8)	159 (91.4)	0.060	287 (94.1)	431 (96.2)	0.178
Need any medical treatment to function in your daily life	50 (14.4)	23 (23.5)	8 (9.3)*	40 (23.0)	0.006	65 (21.3)	63 (14.0)	0.009
Has enough energy for everyday life	63 (18.1)	34 (34.7)	22 (25.6)	91 (52.3)	0.000	127 (41.6)	101 (22.5)	0.000
Ability to get around	19 (5.5)	9 (9.2)	5 (5.8)	18 (10.4)*	0.175	34 (11.2)	22 (4.9)	0.001
Satisfaction with sleep	68 (19.7)	31 (31.6)	29 (33.7)	71 (40.8)	0.000	125 (41.0)	87 (19.5)	0.000
Satisfaction with the ability to perform the daily living activities	26 (7.5)*	20 (20.4)	12 (14.0)	47 (27.0)	0.000	66 (21.3)	46 (10.3)	0.000
Satisfaction with the capacity for work	21 (6.1)*	15 (15.5)*	8 (9.4)*	43 (24.7)	0.000	55 (18.0)	41 (9.2)	0.000
***Psychological domain***								
How much enjoys life	140 (40.5)*	42 (43.3)*	31 (36.5)*	90 (52.0)*	0.040	154 (51.2)*	169 (38.1)*	0.001
Feeling that life has meaning	16 (4.6)*	8 (8.2)	4 (4.7)	18 (10.3)	0.070	25 (8.2)	25 (5.6)*	0.159
Ability to concentrate	83 (24.1)*	30 (30.6)	27 (32.5)*	81 (46.6)	0.000	118 (38.8)*	118 (26.6)*	0.000
Ability to accept your bodily appearance	55 (15.8)	15 (15.3)	16 (18.6)	49 (28.2)	0.006	71 (23.3)	70 (15.6)*	0.008
Satisfaction with oneself	24 (6.9)	14 (14.3)	18 (20.9)	52 (29.9)	0.000	58 (19.0)	56 (12.5)	0.014
Frequency of negative feelings, such as blue mood, despair, anxiety and depression	336 (96.6)	93 (94.9)	82 (96.5)*	157 (90.2)	0.019	281 (92.4)*	429 (96.0)*	0.034
**Social domain**								
Satisfaction with personal relationships (friends, parents, acquaintances, colleagues)	19 (5.5)	8 (8.2)	10 (11.6)	27 (15.5)	0.002	35 (11.5)	32 (7.5)	0.039
Satisfaction with sex life	54 (15.6)*	12 (12.2)	16 (18.6)	16 (17.8)	0.590	55 (18.0)	67 (15.0)*	0.272
Satisfaction with support received from friends	33 (9.5)	11 (11.2)	16 (18.6)	28 (16.1)	0.040	38 (12.5)	55 (12.2)	0.932
***Environmental domain***								
Feels safe in daily life	98 (28.4)*	34 (34.7)	30 (35.3)*	87 (50.0)	0.000	140 (46.1)*	125 (28.0)*	0.000
Quality of the physical environment (climate, noise, pollution, attractive)	222 (64.0)*	69 (70.4)	64 (74.4)	139 (79.9)	0.002	229 (75.1)	303 (67.6)*	0.028
Availability of sufficient money to meet the needs	291 (84.1)*	82 (83.7)	74 (86.0)	147 (84.5)	0.970	270 (88.5)	366 (81.9)*	0.013
Availability of information that is needed in day to day	145 (41.9)	44 (44.9)	48 (57.1)	104 (59.8)	0.000	182 (59.9)*	184 (41.3)*	0.000
Opportunity for leisure activity	252 (72.4)	73 (74.5)	63 (73.3)	138 (79.3)	0.394	258 (84.6)	305 (67.9)	0.000
Satisfaction with the conditions of the living place	83 (23.9)*	25 (25.5)	22 (25.6)	65 (37.4)	0.012	97 (31.8)	116 (25.9)	0.077
Satisfaction with the access to health services	96 (27.6)	36 (36.7)	36 (41.9)	72 (41.4)	0.004	125 (41.0)	133 (29.6)	0.001
Satisfaction with the transport.	116 (33.3)	33 (33.7)	36 (41.9)	73 (42.0)	0.162	120 (39.3)	156 (34.7)	0.198

^ϕ^The frequency of poor quality of life was obtained by the sum of workers with negative responses to each question. The frequency of poor quality of life is shown in the table. What is missing to complete the 100% in the column for each question, considering the total number of workers in each category of the variable effort/reward unbalance and overcommitment refers to workers with good quality of life.

*Absence of answer to the WHOQOL-bref question. ^¥^Chi-square test.

The chance of general poor quality of life was significantly higher among workers with high effort/high reward and with high effort/low reward. It was also higher among workers who reported lower levels of physical activity, those with a negative self-perceived health and common mental disorders, and those who reported systemic disease diagnosed by a physician (Table 2).

**Table 2:** Adjusted model of factors associated with general poor quality of life*

**Variables**	**Prevalence of general poor quality of life (n = 110)**	**OR**_**adjusted**_^**¥ **^**(95% CI)**
***Effort-reward imbalance***		
Low Effort, High Reward	10.1	1
High Effort, High Reward	21.4	2.55 (1.32-4.93)
Low Effort, Low Reward	18.6	1.94 (0.93-4.01)
High Effort, Low Reward	21.8	1.91 (1.07-3.42)
**Individual characteristics**		
***Years of study***		0.92 (0.86-0.99)
**Lifestyle**		
***Physical activity***		
Very active	12.9	1
Active	14.6	1.33 (0.76-2.31)
Irregularly active + sedentary	20.6	2.00 (1.06-3.81)
**Health condition**		
***Self-perceived health x presence of common mental disorders***		
Positive, Absent	20.4	1
Positive, Present	40.7	1.64 (0.94-2.85)
Negative, Absent	58.6	2.40 (0.90-6.42)
Negative, Present	75.0	4.58 (1.78-11.81)
***Presence of systemic disease***		
No	11.5	1
Yes	23.7	1.96 (1.23-3.11)

^¥^Final model adjusted by sex, age and smoking habit. Hosmer-Lemeshow *Goodness of fit*: X^2^ = 4.846; p value = 0.774. *General poor quality of life was defined by the general question “How do you classify your quality of life”. Montes Claros, 2010.

There was a greater likelihood of poor quality of life in the physical and environmental domains among workers who had high effort/low reward, and in the social domain among workers with low effort/low reward, compared to those with low effort/high reward. Workers with high overcommitment had a higher likelihood of poor quality of life in the physical and environmental domains (Tables 3 and 4).

**Table 3:** Adjusted model of factors associated with poor quality of life in the physical and psychological domains

	**Physical domain**		**Psychological domain**	
	**Prevalence of poor quality of life**	**OR**_**adjusted**_^**1 **^**(95% CI)**	**Prevalence of poor quality of life**	**OR**_**adjusted**_^**2 **^**(95% CI)**
***Effort-reward imbalance***				
Low Effort, High Reward	26.7	1	**-**	**-**
High Effort, High Reward	35.7	0.93 (0.53-1.63)	-	**-**
Low Effort, Low Reward	39.6	1.28 (0.72-2.27)	-	**-**
High Effort, Low Reward	54.6	1.65 (1.04-2.66)*	-	**-**
***Overcommitment***				
Low	29.0	1		
High	49.2	1.51 (1.03-2.19)*	**-**	**-**
**Individual characteristics**				
***Sex***				
Male	-	-	15.7	1
Female		-	27.5	2.04 (1.20-3.46)**
***Age***	-	-	-	1.04 (1.01-1.06)**
***Years of study***	-	-	-	0.90 (0.86-0.96)***
***Marital status***				
With partner	36.9	1	-	
Without partner	37.5	1.46 (1.00-2.13)*	-	
**Job characteristics**				
***Job satisfaction***				
Satisfied	22.1	1	18.1	1
Moderately satisfied	39.2	2.12 (1.32-3.38)***	25.2	1.68 (1.02-2.77)**
Dissatisfied	50.6	2.32 (1.30-4.14)**	33.3	1.96 (1.10-3.51)**
**Health condition**				
***Self*-*perceived health x presence of common mental disorders***				
Positive, Absent	27.0	1	18.2	1
Positive, Present	64.4	3.91 (2.48-6.17)***	38.5	2.87 (1.82-4.52)***
Negative, Absent	72.4	3.97 (1.45-10.90)***	51.7	2.70 (1.09-6.70)**
Negative, Present	60.7	1.63 (0.67-3.95)	57.1	4.96(1.96-2.59)***
**Biological function**				
***Presence of systemic disease***				
No		1		
Yes	0.000	1.61 (1.10-2.35)**	-	-

^1^Final model adjusted by sex and age. Hosmer-Lemeshow Goodness of fit: X^2^ = 2.568; p value = 0.958. ^2^Final model adjusted by marital status and presence of systemic disease. Hosmer-Lemeshow *Goodness of fit*: X^2^ = 5.755; p value = 0.68. * < 0.05; ** < 0.01; *** < 0.001.

**Table 4:** Adjusted model of factors associated with poor quality of life in the social and environmental domains

	**Social domain**		**Environmental domain**	
	**Prevalence of poor quality of life**	**OR**_**adjusted**_^**1 **^**(95% CI)**	**Prevalence of poor quality of life**	**OR**_**adjusted**_^**2 **^**(95% CI)**
***Effort****-****reward imbalance***				
Low Effort, High Reward	19.3	1	17.8	1
High Effort, High Reward	19.4	0.97 (0.52-1.81)	29.6	1.24 (0.62-2.46)
Low Effort, Low Reward	30.2	1.82 (1.00-3.30)*	27.9	1.69 (0.88-3.27)
High Effort, Low Reward	25.9	1.23 (0.74-2.04)	43.1	2.39 (1.37-4.16)**
***Overcommitment***				
Low	-	-	18.5	1
High	-	-	39.7	1.69 (1.08-2.65)*
**Individual characteristics**				
***Years of study***	-	-	-	0.90 (0.82-0.98)*
***Income***	-	-	-	0.99 (0.98-0.99)**
***Marital status***				
With partner	19.3	1	29.9	1
Without partner	24.5	1.83 (1.21-2.76)**	24.3	0.62 (0.40-0.94)*
**Job characteristics**				
***Work position***				
Superior level	14.8	1		
Technical level	20.0	1.63 (0.80-3.30)	-	-
Middle level	24.8	2.10 (1.23-3.59)**	-	-
***Labour regime***				
Effective			21.5	1
Contracted	-	-	34.2	1.77 (1.09-2.90)*
***Job satisfaction***				
Satisfied			16.1	1
Moderately satisfied	-	-	28.3	2.19 (1.22-3.94)**
Dissatisfied	-	-	38.5	2.75 (1.38-5.46)**
**Lifestyle**				
***Smoking habits***				
Non smoking	21.0	1		
Light or moderate smoker	13.3	0.92 (0.19-4.39)	-	*-*
Heavy smoker	44.4	3.13 (1.38-7.11)**	-	*-*
**Health condition**				
***Self*-*perceived health x Presence of common mental disorder***				
Positive, Absent	18.9	1	19.8	1
Positive, Present	30.4	1.81 (1.12-2.95)**	42.2	2.40 (1.44-3.98)***
Negative, Absent	31.0	1.37 (0.51-3.69)	41.4	2.21 (0.81-6.03)
Negative, Present	35.7	1.22 (0.47-3.21)	78.6	7.45 (2.42-22.87)**

^1^Final model adjusted by sex and age. Hosmer-Lemeshow *Goodness of fit*: X^2^ = 6.323; p value = 0.61. ^2^Final model adjusted for sex, age and presence of systemic disease. Hosmer-Lemeshow *Goodness of fit*: X^2^ = 5.626; p value = 0.689. * < 0.05; ** < 0.01; *** < 0.001.

Also associated with an increased likelihood of poor quality of life in the physical domain were living without a partner, job dissatisfaction, the presence of common mental disorders, negative self-perceived health, and presence of systemic diseases diagnosed by a physician. In the psychological domain, a greater likelihood of poor quality of life was also observed among female workers, the oldest workers, those with fewer years of study, those dissatisfied with their work, and those who had both negative self-perceived health and the presence of common mental disorders (Table 3).

In the social domain, those more likely to exhibit poor quality of life were workers who were living without a partner, workers in the middle levels, heavy smokers, and workers with common mental disorders and systemic disease diagnosed by a physician. In the environmental domain, an increase in the years of study and income of workers was associated with a decrease in the occurrence of poor quality of life. Individuals without a partner were less likely to have poor quality of life in this domain. There was a greater likelihood of poor quality of life among contracted workers, those dissatisfied with their work, those who had common mental disorders, and those who had both common mental disorders and negative self-perceived health (Table 4).

## Discussion

The effect of psychosocial work conditions on the quality of life of health workers was assessed among workers of the Primary Health Care. After adjusting for individual characteristics, job characteristics, and lifestyle and health conditions, there remained a significant association between effort-reward imbalance and poor quality of life, for general quality of life, as well as its physical, social, and environmental domains. Overcommitment was significantly associated with poor quality of life in the physical and environmental domains. Overall, there was a greater prevalence of poor quality of life among workers with effort-reward imbalance (high effort/low reward) and high overcommitment.

This study revealed poor quality of life among 15.4% of workers, a lower percentage than that recorded among dentists in the public service of Paraná, Brazil (26.2%) [19]. However, the frequency of poor quality of life in the four domains was higher among these professionals than among Primary Health Care workers in the present study. The differences between the two studies may be explained by differences in the cut-off point for the definition of poor quality of life. The cut-off point for the definition of quality of life in the study of dentists was the median [19]. In the present study, the lower quartile was chosen to define poor quality of life because those with lower scores more accurately represent individuals in more unfavorable situations. A Brazilian study, with a southern general population sample, demonstrated 25th percentiles scores similar to those obtained in this study for the physical (25th percentile score: 54), psychological (25th percentile score: 58), social (25th percentile score: 67), and environmental (25th percentile score: 50) domains [32]. No Brazilian population studies, or studies with representative samples for the population from which the workers in this study were taken, were found, thus hindering the comparison of the results of this study with other population data, since there is heterogeneity among regions of the country.

Workers with high effort/low reward and/or high overcommitment had a higher prevalence of general poor quality of life. Quality of life can be compromised by adverse psychosocial conditions at work, which has an impact on public and occupational health [17]. Workplace characteristics and the process of working in the Primary Health Care can cause fatigue in workers and risk to their physical and mental health [25]. The organization of work influences the health of workers, enabling or hindering self-control over daily tasks [33]. Teamwork and interaction between individuals with different competencies and abilities is required for the effectiveness of the Primary Health Care and facilitates more comprehensive care to users. This dynamic increases the competition between professionals, as well as the differences between what is prescribed and real work [24]. The Primary Health Care is based on a multidisciplinary approach, and on general practice and generalist knowledge. This increases the uncertainty and tension among workers, also making the coordination of the work process more complex [33,34].

Additionally, the precariousness of health services within the scope of the Brazilian Primary Health Care is a fact, represented by the accumulation of work, uncertain work contracts, insecurity and/or job instability, lack of plans for positions and salaries, and difficulty in exercising labor rights [8,25,34]. These situations can generate fear, isolation, and submission [33].

The prevalence of poor quality of life in the physical, and environmental domains was greater among workers with high effort/low reward and/or high overcommitment. Poor quality of life in in the physical domain was manifested by dissatisfaction with sleep, an inability to perform activities of daily life due to a lack of energy, the need for medical treatment to carry out daily activities, and an incapacity for work. According to the *Effort-Reward Imbalance* model, workers with high effort/low reward experience high physical and psychological demands at work without the benefit of rewards in the form of financial gain (adequate salary), self-esteem (respect and support from colleagues and superiors), and/or safety and opportunities (promotion prospects, job security, and social *status*). Overcommitment is a personal intrinsic component, resulting in a set of attitudes, behaviours, and emotions that reflect excessive stress in combination with a strong desire to be accepted and appreciated. People with overcommitment tend to exaggerate their efforts. There is evidence that excessive stress results in an underestimation of the challenge, which in turn can be triggered by an underlying motivation to try and experience esteem and approval [35]. Still, according to the model, an overcommitted person will respond in a more inflexible manner to situations of high stress at work, with greater stress than another, less overcommitted person in the same situation [35]. A Brazilian study demonstrated a greater probability of poor self-reported health among nurses in public hospitals who exhibited concomitant high overcommitment and effort-reward imbalance (OR = 2.74; 1.88–3.99), compared to those who exhibited only effort-reward imbalance (OR = 2.36; 1.46–3.82), or only overcommitment (OR = 2.04; 1.33–3.14) [36]. In the present study, the components of the *Effort-Reward Imbalance* model were analysed separately to adhere to the predominant manner used in the literature. Thus, it was possible to discuss the factors related to the individual worker separate from those related to the work environment.

Effort-reward imbalance and high overcommitment at work have been considered a risk factor for health, such as cardiovascular disease, musculoskeletal pain, and psychosomatic complaints [4-6], and is associated with sick leave, the intention to retire, or to quit their position [37], therefore justifying its association with the physical domain of quality of life [7,38]. Job strain has been associated with symptoms such as headaches, sore shoulders and neck, bruxism and jaw pain, indigestion, nausea, stomach ulcers, loss of efficiency at work, and insomnia, among others [39]. Studies that have investigated the relationship between quality of life and adverse psychosocial work conditions (assessed by the *Effort-Reward Imbalance* model) among Primary Health Care workers have not been identified. There is a consistency between the results of this study and those studies involving health workers [18] or workers from other areas [40]. The effort-reward imbalance was a significant predictor of the physical and psychological functions of quality of life in a longitudinal study among public British workers [40]. In a longitudinal study of German workers [41], work ability and physical function were negatively affected by the effort-reward imbalance, analysed as the ratio between the scores of effort divided by the reward score > 1 (*Sociomedical Panel of Employees*). In this longitudinal study [41], the assessment of work ability had similar characteristics to those that constitute the physical domain of quality of life, such as current work ability and the inability to work due to illness. The physical function, as assessed by the instrument *Short-Form Health Survey* (SF-36), includes items regarding limitations in performing activities of daily life, similar to those comprising the physical domain of the WHOQOL-BREF.

There was no association between effort-reward imbalance and overcommitment with quality of life in the psychological domain. This result was surprising, since the psychological consequences of stress at work are discussed in the literature [5,7]. In this study, this association may have lost significance in the multiple regression model that was adjusted according to self-perceived health and the presence of common mental disorders (anxiety and depression). The facets of the psychological domain suggest that these two variables can be interpreted as part of the response variable (psychological domain of quality of life), with common variance, making them hardly distinguishable. All these measures are subjective because they relate to the worker’s own assessment of his health condition. Workers who had isolated or concomitant negative self-perceived health status and mental disorders showed a 3–5 times greater probability of reporting poor quality of life. A systematic review, based on prospective studies, concluded that there is evidence of the effect of psychosocial risk factors at work, such as effort-reward imbalance, for the development of mental disorders related to stress [7]. In a study of workers in the banking sector, exposure to adverse psychosocial conditions at work, as measured by the *Effort-Reward Imbalance* model, was independently associated with poorer self-perceived health [14]. One hypothesis is that individual characteristics (sex, age and years of study) and job satisfaction are stronger determinants of psychological quality of life in this population of Primary Health Care workers. The poor quality life in this domain was manifested by the amount that the individual enjoys life, your ability to concentrate, your ability to accept you bodily appearance, and satisfaction with oneself. This feeling can to be worst in female, older and in those with lower education workers.

In the social domain, workers with low effort/low reward demonstrated a greater prevalence of poor quality of life compared with those with low effort/high reward. In this domain, low reward was revealed as a determinant of poor quality of life, manifested in dissatisfaction with the support received from friends and personal relationships. In the *Effort-Reward Imbalance* model, reward includes respect and support from work colleagues and superiors, the feeling of being treated fairly and/or promotion prospects, job security, and social status. The results suggest that work relations interfere in the perception of an individual’s personal relationships; or those who have problems in relationships in the workplace can also more frequently exhibit problems in other areas of life. However, interpersonal relationships in the workplace have been identified as a challenge in the Primary Health Care. Among the workers of the Primary Health Care in Federal District, Brazil, the socio-professional relationships in teams presented the most serious situation in the work process. The authors noted difficulties in communication between management and subordinates, lack of integration in the workplace, poor communication among staff, and lack of support from management for professional development [25]. Such a scenario affects the ‘individual and collective process of rewriting the norms, the rules, which can harm the health of the worker, as well as the quality and effectiveness of the service provided to the population’ [25].

Workers with high effort/low reward and high overcommitment had a higher prevalence of poor quality of life in the environmental domain, revealed by feelings of insecurity on a daily basis, an unhealthy physical environment, a lack of access to information, less opportunity for leisure activities, dissatisfaction with where they live, and less access to health services. This result may reflect job insecurity, work overload, lack of professional development and inadequate working conditions. Such conditions can affect people's perception of life and the environment where they live. Related to this finding was the observation made by Primary Health Care workers of the Federal District, Brazil, that working conditions were negatively affected by discomfort in the physical environment, the existence of noise in the workplace, the inadequacy of the workplace for the completion of tasks, and by people’s insecurity [25]. Of the independent variables related to working conditions considered in this study, two were also associated with poor quality of life in the environmental domain: job dissatisfaction and labour regime. Dissatisfied and contracted workers may feel more vulnerable due to the possibility of job loss, compromising their health and quality of life. Lack of job stability and insufficient wages preclude the realization of commitments, the availability of time and resources for health care, and the use of media to access information. Thus, the demands of work become incompatible with a fulfilling family life, consequently leading to insecurity in daily life. Unstable job regimes are considered a major factor in the high turnover and dissatisfaction of Primary Health Care workers [42].

As previously noted in the literature, numerous variables were associated with an increased prevalence of poor quality of life in general, and in the four domains, including individual characteristics and job characteristics, lifestyle and general health variables: increased age [43], income [44], being female [20]; education level [45], living without a partner [39], being dissatisfied at work, being middle level and contracted [46], having lower levels of physical activity and smoking heavily [47], negative self-perceived health, having common mental illnesses [48], and having diseases diagnosed by a physician. The variables associated with each domain provide evidence for their different constructs and determinants.

One limitation of this study is that its data are based on subjective evaluations. Some authors suggest that using the same method to assess the independent and dependent variables leads to an inflated correlation. This situation can be explained by negative affectivity, which is a personality trait that predisposes individuals to experience negative emotions, leading to pessimism about the world. Thus, these people tend to consider their living conditions worse, their job more stressful, and their quality of life more affected than others without negative affectivity [49]. In addition, reverse causality cannot be ruled out, since individuals with poor quality of life may overestimate their psychosocial stress at work. Moreover, studies based on these measures may be influenced by factors such as recall bias and socially desirable responses. In future studies other variables related to the context and work process in the Primary Health Care should be considered, allowing for greater understanding of the associations in an actual work environment.

This study indicates the need to investigate public policies promoting employee health, programs for managing stress in the workplace, and professional development. The quality of life of Primary Health Care workers may indicate the quality of services offered by health institutions, and for this reason, investing in the quality of life of those who care for other people in the Unified Health System can bring benefits to not only to workers, but also its users.

## Conclusion

Adverse psychosocial conditions at work, represented by effort-reward imbalance and high overcommitment, are associated with poorer quality of life among Primary Health Care workers. Moreover, quality of life was determined by individual characteristics, job characteristics, lifestyle, and health conditions of the workers. This result confirms the multifactorial construct of the quality of life. Actions aimed at improving the health and quality of life of workers must have intersectoral nature taking into account the adverse psychosocial working conditions.

## Competing interests

The authors have no financial or non-financial competing interests with any people or organizations.

## Authors’ contributions

MABT contributed to acquisition of data, and was the main writer of the paper. MRB participated in the literature review and drafting of the article. AMDV contributed to analysis and interpretation of data. VEG e EFF contributed to writing the article and revised the final version. AMEBM contributed to conception and design, and interpretation of data. RCF completed the statistical analysis, contributed to the drafting of the article, and revised the final version. All authors read and approved the final manuscript.
